# Supplementary material for: MetaRibo-Seq measures translation in microbiomes
Source: Nat Commun. 2020 Jun 29;11:3268. doi: 10.1038/s41467-020-17081-z (PMC7324362; doi:10.1038/s41467-020-17081-z)
Supplement: Supplementary file 10 — Supplementary Data 7 [file 41467_2020_17081_MOESM10_ESM.zip › File2/Confidence_High_Taxonomy/306669_out.krona.html]

Javascript must be enabled to view this page.

members
magnitude
magnitudeUnassigned
count
unassigned
taxon
rank

306669\_out

4

4
2
superkingdom

4
976
phylum

class
4
200643

171549
4
order

family
4
815

genus
4
816

species
4
310297

SRS013476\_contig\_number\_contig-100\_669.225759SRS024132\_contig\_number\_21027SRS024140\_contig\_number\_contig-100\_2060.2060SRS078419\_contig\_number\_22704
